# Development and Validation of a Questionnaire on Consumer Psychological Capital in Food Safety Social Co-governance

**DOI:** 10.3389/fpsyg.2020.584810

**Published:** 2021-01-07

**Authors:** Chun Meng, Lin Sun, Xiaoni Guo, Miao Wu, Yuqi Wang, Lingping Yang, Bin Peng

**Affiliations:** School of Public Health and Management, Chongqing Medical University, Chongqing, China

**Keywords:** food safety social co-governance, consumers, psychological capital, questionnaire development and validation, reliability test, confirmatory factor analysis

## Abstract

Consumers play an important role as one of the main actors in food safety social co-governance. To create a pattern of food safety social co-governance, the active and effective participation of consumers is critical. To encourage consumers to participate in food safety social co-governance voluntarily and positively, we attempted to develop and preliminarily validate a multidimensional questionnaire on consumer psychological capital that could be used to measure the degree of consumer participation in food safety social co-governance. The aim of the initial sample (*N* = 170) and test sample 2 (*N* = 204) was to investigate the factor structure of a preliminary measure of consumer psychological capital. A 4-factor model with 23 items explained 61.05% of the total variance in item scores. The aim of test sample 3 (*N* = 30) was to measure the retest reliability. Test sample 4 (*N* = 1,076) was randomly allocated to the modeling sample (*N* = 538) and validation sample (*N* = 538) to verify questionnaire reliability and validity. Convergent validity, discriminant validity, and the internal inconsistency coefficients of the questionnaire were assessed in the modeling sample. While processing CFA, we deleted 9 items with small standardized factor loadings. The remaining 14 items in the final revised 4-factor model included self-efficacy, resilience, hope, and optimism. The fit indices of the revised four-factor model and second-order factor model in the modeling sample revealed an acceptable model fit. The convergent validity and discriminant validity of the revised model were good and acceptable, respectively. A cross-validation procedure confirmed the appropriateness of the revised four-factor model and second-order factor model in the validation sample. The cross-validation results confirmed that the fit indices of the revised four-factor model fitted the data well and the second-order factor model in the validation sample reached acceptable values. We concluded that the questionnaire developed in this study had good reliability and stable and acceptable construct validity. It could provide a theoretical basis for measuring psychological capital in food safety co-governance.

## Introduction

Food safety is a topic of central interest to almost all members of society as it is particularly pertinent to health. In China, the government previously played a dominant role in regulating and monitoring food safety (Liu et al., 2019). Excessive government intervention suppressed the market’s regulatory role, and safety incidents still occur frequently (Wang et al., 2018; Yi et al., 2019). In this circumstance, more effective approaches to food safety governance must be explored. Co-governance, as a transparent and effective approach, is practiced extensively in developed countries (Kelnert and Silva, 1993). Co-governance combines government regulation and social self-governance and is the extension and development of a unitary government governance mechanism (Fairman and Yapp, 2010). Compared with vertical, top-down, one-way government governance mechanisms, co-governance has become the basic form of food safety governance worldwide for guaranteeing food safety with more efficient governance allocation and lower costs (Martinez et al., 2007). To improve the efficiency of food safety governance, reduce governance costs, and reduce or even eliminate food safety risks (Coglianese and Lazer, 2003; Martinez et al., 2007), in June 2013, the government introduced the concept of social co-governance for food safety, which consists of five parts: government supervision, enterprise autonomy, social cooperation, public participation, and legal protection (Wu et al., 2018). The Food Safety Law of China enacted in October 2015 legislated social co-governance as the basic principle of food safety management in China (State Administration for Market Regulation, 2015). The rights and freedom of government, enterprises, and social actors are equal in social co-governance, which is different from traditional governance activities (Martinez et al., 2007). In the food safety social co-governance process, diverse actors cooperate and work together to regulate food safety at a lower cost by the combined and synergetic use of multiple instruments, such as government regulation, market incentives, technical regulation, social supervision, and information dissemination under the framework of laws and regulations, to ensure a higher level of food safety and achieve maximum social welfare (Martinez et al., 2007; Wu et al., 2018).

The fundamental cause of food safety problems lies in the information asymmetry between food manufacturers and consumers, which could lead to market failure (Wu et al., 2018). If the government fails to address the market failure, it probably appears as a “double failure” of government public right and market private right in the process of food safety management (Chen and Wu, 2019). Thus, it is critical for consumers to employ their unique advantages in food safety co-governance actively and effectively. The consumer is one of the main actors of food safety social co-governance. Moreover, consumers are not only the most immediate beneficiaries of food safety but also the most direct sufferers of unsafe food. This means they have strong incentives to address food safety issues and protect their health (Chen and Wu, 2019) and to contribute to the social co-governance of food safety (Rouvière and Caswell, 2012). However, consumers have been criticized for lacking consciousness to voluntarily supervise food safety issues (Mohd Nawi and Mohd Nasir, 2014; Ren et al., 2015) and lacking knowledge of the appropriate channels for making food safety complaints (Cheng et al., 2017). The whistleblowers who reported the food safety issues failed to get timely responses (Yin et al., 2015) or adequate privacy protections and reporting rewards (Zhang, 2013). However, several efforts have been made to mobilize consumer participation in food safety social co-governance. For example, some scholars have recommended the government establish a public interest litigation mechanism similar to that in the United States, which covers penalizing illegal enterprises, rewarding consumers who report illegal activities, and encouraging consumers to participate in food safety co-governance (Yin et al., 2015; Li et al., 2016). Other researchers have argued that the government should provide appropriate rewards and adequate protections for whistleblowers (Moy, 2018) and establish an information disclosure system so that consumers can acquire more food safety information and better understand how to participate in food safety governance (Boumil et al., 2010). Vukatana et al. (2016) urged for improving the transparency and accessibility of the food safety system, which could significantly improve consumers’ regulatory capabilities. It seems that these food safety co-governance policies do not work well for consumers, indicating that the value and effectiveness of these food safety co-governance policies need to be demonstrated in future practice. Therefore, the purpose of this article is to explore how to improve consumer eagerness to participate in food safety co-governance from a non-policy perspective, so that consumers can voluntarily play the supervisory role in food safety social co-governance.

Without taking advantage of psychological capital’s role in the food safety management process, co-governance alone is not sufficient (Chen and Wu, 2019). Luthans and Youssef-Morgan (2004) proposed the concept of psychological capital consisting of four core components: self-efficacy, optimism, resilience, and hope within the framework of positive psychology (Seligman and Csikszentmihalyi, 2000) and positive organizational behavior (Luthans, 2002). These four structures not only have conceptual independence and empirically based discrimination validity but also promote each other and work synergistically, thereby forming a higher-level construct of psychological capital (Luthans et al., 2007a). The 24-item psychological capital questionnaire developed by Luthans et al. (2007b) is mainly applicable to staff and managers, and its applicable objects are relatively limited. In addition, the questionnaire lacks sufficient evidence of validity, so it is not suitable for measuring consumer psychological capital in the social co-governance of food safety. Chen and Wu (2019) were the first scholars to apply the concept of psychological capital to the social co-governance of food safety. They stated that promoting consumer positive psychological capital enhanced consumer confidence and enthusiasm for participating in food safety co-governance. However, there is a lack of empirical studies investigating the role of consumer psychological capital in food safety co-governance.

To summarize, this paper mainly developed and validated a scale to measure consumer psychological capital in food safety social co-governance from a non-policy perspective and encouraged consumers to play the role of supervisor voluntarily and positively. Our quantitative study broadens the research scope of psychological capital and provides a novel tool to measure consumer participation in food safety social co-governance.

## Materials and Methods

### Materials

#### Literature Review

China has long implemented a single governance structure of food safety supervision, which is mainly supervised by government departments and supplemented by the supervision of food categories (Liu et al., 2019). With the transformation of economic and social structures and the development of market economy, the diversity of food types and food supply abundance make food quality and safety a common societal concern and bring great challenges to food safety supervision. A series of food safety scandals in recent years have reflected the inability of traditional regulatory systems to adapt to society’s need for safe food (Wang et al., 2018; Yi et al., 2019). The government urgently needs to find more effective approaches to food safety governance in response to public expectations, for public confidence in the government’s ability to manage food safety risks was diminished and shaken (Halkier and Holm, 2006).

In the late twentieth century, the government’s regulatory capacity lagged behind social development in food safety issues (Wu et al., 2018). At the same time, social organizations and citizen groups greatly promoted the development of social governance. The government had to coordinate and cooperate with social actors in many ways (James, 1992). The co-governance theory was gradually accepted by managers and researchers. By the end of the twentieth century, the theory of social governance emphasizing the construction of a collaborative network that enables multilateral interaction between diverse, decentralized actors began to blossom (Unwin, 1995). A flexible and inclusive concept of social co-governance, which is a new form of co-governance theory in the process of social development, was formed. The theory of social co-governance was one of the most influential theories in the field of public administration at that time. The diversity of participating actors is the core of this theory, which breaks away from the traditional single mode of government governance and thus reflects the publicity in the field of administration. They exercise respective powers according to the provisions of the law to jointly manage public affairs to realize the common interests of the entire society. Given the importance of preventing food safety risks, researchers have extended the concept of social co-governance to food safety governance (Martinez et al., 2007). Food safety social co-governance is the process by which the government and social organizations coordinate and cooperate in setting food safety standards, process implementation, enforcement, and monitoring to provide higher quality and safer food at a lower governance cost (Martinez et al., 2007). Noticeably, actors involved in food safety co-governance are equal partners, unlike in traditional governmental governance activities (Wu et al., 2018).

Psychological capital forms the basis of prosperity and happiness because it ignites positive emotions and feelings of appreciation. Self-efficacy is the self-confidence that an individual is competent for tasks, can face challenges and can strive to succeed (Parker and Sharon, 1998). Optimism is held by individuals who have a positive attribution style and a positive attitude toward the present and the future (Luthans, 2002). Resilience is an individual’s ability to quickly recover from adversity, setbacks and failures, and even to actively change and grow (Masten and Ann, 2001). Hope is a state of positive motivation that strives to achieve a predetermined goal through various means (Luthans et al., 2007a). It is necessary to fully recognize the role of psychological capital in stimulating the vitality of consumers and promoting consumer participation in food safety social co-governance (Chen and Wu, 2019). From the perspective of psychological capital, consumers, as beneficiaries of food safety, have a strong motivation to improve food safety and ensure their health. Our quantitative study proposed two hypotheses: (1) Consumer psychological capital set as the second-order factor could explain the four factors including self-efficacy, optimism, resilience, and hope. (2) There are positive correlating relationships between the four factors. If the two hypotheses are true, we can investigate how consumer psychological capital affects consumer participation in future research so that we can make efforts in a more specific direction to improve and encourage consumer enthusiasm to participate in food safety social co-governance.

#### The Formation of the Initial Questionnaire

The four-dimensional psychological capital concept (Luthans and Youssef-Morgan, 2004)—comprising self-efficacy, resilience, hope, and optimism—was adopted in developing the questionnaire to measure consumer psychological capital. Through a literature search (in Chinese and English), we retrieved questionnaires relating to the four factors of psychological capital, analyzed the dimensions of psychological capital these questionnaires measured, then produced the questions, and finally constructed a pool of items that reflected the four dimensions of consumer psychological capital. Three experts—a psychologist, an epidemiologist, and a biostatistician—and three graduate students in psychology independently evaluated the validity of the items for each dimension in the pool. After a discussion within our research group, three levels of items with evaluations of very effective, effective, and average were selected. The initial questionnaire consisted of 39 items, with 9–10 items for each dimension. For example, “I can positively learn about ways to protect food safety through mobile phones, TV, or offline publicity.”, “If I buy expired, spoiled, moldy, or poisonous food, I will inform the people around me in time.”, “If I report a problem with food, I hope to receive a timely and fair response from the relevant department.”, and “I believe that most of the foods circulating in the market are safe.”. The items were scored on a seven-point Likert-type scale (ranging from 1 = strongly disagree to 7 = strongly agree); the higher was the score of the items, the higher the psychological capital.

#### Quality Control of the Questionnaire

The respondents were required to answer all items in the survey to ensure data completeness. They were allowed to fill in the questionnaire only once via WeChat to prevent duplicate questionnaire responses. Questionnaires with 20 consecutive identical answers were discarded, as were initial questionnaires with a response time of less than 100 s and formal questionnaires with a response time of less than 300 s.

### Methods

#### Sample and Test Procedure

Our study administered the preliminary and formal questionnaires, using the Questionnaire Star platform, which is an online professional questionnaire survey platform. All surveys were sent to consumers who met the following criteria: (1) they were capable of using the internet to fill in the online questionnaire; (2) they were literate; (3) they could take part in the survey voluntarily; (4) they were 18 or older; and (5) they had experience buying food. Consumers over the age of 18 were informed about the study’s purpose, privacy protection and anonymity before completing the electronic questionnaire. Informed consent was given on the first page of the online survey. The ideal sample size for a preliminary survey should be 5–10 times the total number of the items included in the questionnaire (Comrey and Lee, 1992), whereas for the final survey, the sample size should be 40–50 times the total number of items (Cummings et al., 1988; Comrey and Lee, 1992; Price, 1993). The first and second preliminary surveys had sample sizes of 188 and 249, respectively. The final survey had a sample size of 1,307. From the total of 188 consumers surveyed to test the initial questionnaire, 170 valid questionnaires were obtained (initial test sample, effective rate = 90.43%). Items were deleted if they had a discrimination degree of less than 0.4 or their factor loadings did not meet the single-dimension requirement. After adding a few items from the pool and adjusting the presentation of some items, we constructed a retest questionnaire with a total of 30 questions across the four dimensions of psychological capital. The initial 30 items of test sample 2 are presented in Table 1.

**TABLE 1:** The initial 30 items in test sample 2.

Self-efficacy	Q4. I can positively learn about ways to protect food safety through mobile phones, TV, or offline publicity.
	Q5. I will pay attention to the food safety incidents released by the news media and take the initiative to stay away from fake and inferior food.
	**Q6**. If I buy expired, spoiled, mildewed, or toxic food, I can quickly complain and report it to the relevant department by telephone, letter or network, etc.
	Q7. I know how to find online channels and methods for safeguarding consumer rights.
	Q8. I can use QQ, WeChat, Weibo, and other network platforms to play the role of a food safety supervisor better.
	**Q9**. If I find that illegal businesses have illegal operations, I dare to report it to the relevant departments.
	**Q10**. If I encounter a serious food safety issue such as food poisoning, I can report it to the relevant department in time.
	**Q11**. I think I have a strong sense of responsibility for food safety.
Resilience	Q12. I have zero tolerance for unsafe and unqualified food.
	**Q13**. If I find out that food has expired or deteriorated before buying it, I will take the initiative to submit it to the merchant.
	Q14. If I buy expired spoiled food and look for compensation from businesses but rejected, I will choose to continue to complain, and inform to maintain rights and interests.
	Q15. If someone tells me that I’m meddling when I report food that has expired, gone bad, gotten mildewy, or is toxic, I will still insist on speaking up.
	**Q16**. If I buy expired, spoiled, moldy, or poisonous food, I will inform the people around me in time.
	Q17. If I buy food that is expired, spoiled, moldy or poisonous, I will learn from experience and improve my food safety awareness.
	Q18. If I eat out or order takeout and eat unhygienic food, I will inform the business and get a reasonable solution.
	**Q19**. When buying food, I will try to understand its safety as much as possible by checking the shelf life, color, and taste.
Hope	Q20. If I report problematic food, I hope the responsible businesses will be punished accordingly.
	Q21. If I report a problem with food, I hope to receive a timely and fair response from the relevant department.
	Q22. I think participating in food safety co-governance can not only protect your legal rights and health but also protect the rights and health of other consumers.
	**Q23**. I think that I have a sense of social responsibility, prompting me to participate in food safety co-governance and give full play to food safety supervision.
	**Q24**. I think that active participation in food safety co-governance and being a good food safety supervisor is beneficial for the food safety social co-governance.
	**Q25**. I understand that I have the most personal experience of safe food and should actively participate in food safety co-governance.
Optimism	Q26. I believe that most of the foods circulating in the market are safe.
	Q27. I believe that most consumers can play a role in food supervision.
	Q28. I believe that most consumers can positively report, complain about unsafe food that they encounter.
	**Q29**. For manufacturers or brands that have exposed food safety incidents, I believe they can take corresponding responsibilities and correct them.
	**Q30**. I believe that most food business operators have an attitude of safety-first and benefit second.
	**Q31**. I believe that the media can accurately and timely disseminate food safety incidents.
	**Q32**. I think food regulators will promptly deal with food safety issues reported by consumers.
	Q33. In my opinion, it would be effective for the government, enterprises and society to jointly deal with food safety issues, instead of relying solely on government supervision.

*14 items in bold were left in the final questionnaire: self-efficacy (Q6, Q9, Q10, Q11); resilience (Q13, Q16, Q19); hope (Q23, Q24, Q25), and optimism (Q29, Q30, Q31, Q32).*

Subsequently, we surveyed 249 consumers to test the retest questionnaire and obtained 204 valid questionnaires (test sample 2, effective rate = 81.93%). Based on the results of the item analysis and exploratory factor analysis (EFA), items whose factor loadings did not meet the single-dimension requirement were excluded. Finally, we constructed the formal questionnaire consisting of demographic information and 23 questions across four dimensions of psychological capital (Supplementary Table S1). To assess the formal questionnaire’s retest reliability, 30 consumers were recruited to complete the formal questionnaire twice within 2 weeks (test sample 3). Then, 1,307 consumers were enrolled to complete the formal questionnaire, and 1,076 (test sample 4, effective rate = 82.33%) of them returned valid questionnaires. Test sample 4 included 370 men and 706 women aged between 18 and 76 years old. Confirmatory factor analysis (CFA) was used to test the rationality of the four-factor model, and the test level was defined as alpha = 0.05.

#### Statistical Analysis

The critical value ratio, correlation, internal consistency, and reliability of the questionnaire were calculated. EFA was carried out by using SPSS version 22 on the initial test sample and on test sample 2 to derive the formal questionnaire. The extreme group method was used to test the identification degree of the items. Items with a determination value of less than 3 and *p* value greater than 0.05 were deleted (Wu, 2000). If the correlation coefficient between each item score and the total score of all items was less than 0.4, the item was deleted (Giacomin et al., 2008). Items with a factor loading less than 0.4 were also excluded from the questionnaire (Markus, 2012). If deleting an item caused the Cronbach’s alpha coefficient to increase significantly (greater than the Cronbach’s alpha coefficient of the questionnaire), this item was also removed (Wu, 2000).

In test sample 4, the survey select procedure in SAS version 9.4 was used to ensure random sampling (seed = 1,234). Test sample 4 was randomly separated into a modeling sample and validation sample, each with a sample size of 538. The modeling sample was used to evaluate the factor structure of the CFA, while the validation sample was used for the cross-validation procedure. Cronbach’s alpha coefficient (modeling sample) and the retest reliability coefficient (test sample 3) were calculated to assess the questionnaire’s reliability. Cronbach’s alpha coefficients of the questionnaire greater than 0.80 and 0.70 indicated good, and acceptable internal consistency, respectively (Robinson et al., 1991; DeVellis, 2003; Hair et al., 2010).

CFA was conducted to examine the validity of the model structure formed by EFA. The models were estimated using maximum likelihood estimation. In the basic CFA model, one non-standardized factor loading from each factor and the non-standardized regression coefficients of the 23 residuals were set as 1. The CFA consisted of three steps: (1) evaluating the conceptual factor structure; (2) modifying the factor structure and improving the model fit; and (3) cross-validation. We conducted the CFA on the modeling sample using AMOS version 17.0. To make the model fit the sample data, the items with low standardized factor loadings were deleted in the model modification procedure. We also freed the residual correlation within the same factor according to the modification index (MI). The CFA was then conducted on the validation sample to assess the stability of the factor structure. χ^2^/df ratios (Hair et al., 2010), the root mean square error of approximation (RMSEA) (McDonald and Ho, 2002), standardized root mean square residual (SRMR) (Medsker et al., 1994; Hu and Bentler, 1999), comparative fit index (CFI), and Tucker–Lewis index (TLI) (Tucker and Lewis, 1973; Bentler and Bonett, 1980) were used in model selection.

Convergent validity confirms that the scale is correlated with other known measures of the concept. Construct reliability (*CR* ≥ 0.70), standardized factor loadings (0.50–0.95 with a significance level of 0.05), and average variance extracted (AVE ≥ 0.50) (Bagozzi, 1981) were applied to ensure excellent convergent validity. Discriminant validity ensures that the scale is sufficiently different from other similar concepts. CFA provides two common ways of assessing discriminant validity. First, a square root of AVE for any two constructs is greater than the correlation estimate between these two constructs, indicating high discriminant validity (Hair et al., 2010; Nagase and Kano, 2016). This is a rigorous way to test because even a small correlation between latent constructs would imply a lack of discriminant validity (Segars, 1997). In this exploratory research, the second loose way was referenced, we performed chi-square tests on nested models to support the discriminant validity of the revised model, with a statistical significance level of *p* < 0.05.

## Results

### Item Analysis

A total score of psychological capital items for each consumer was calculated, and all scores were ranked in descending order. Consumers who were ranked in the top 27% (scores: 182–209) and the bottom 27% (scores: 106–151) were defined as having high and low psychological capital, respectively. The *t*-value of the items ranged from 6.15 to 13.47 in the independent samples *T*-test, all of which met the statistical significance level of *p* < 0.05. A Kolmogorov–Smirnov normality test on the total score (*p* > 0.05) and the score of each item (*p* < 0.05) suggested that the Spearman’s rank correlation should be used to assess the correlation between each item and the total score. The correlations were between 0.50 and 0.71 with a *p*-value lower than 0.05. The factor loadings were between 0.47 and 0.70. As a result, 30 items in the test sample 2 questionnaire were retained (see Table 1). The results of the item analysis are shown in Table 2.

**TABLE 2:** Item analysis in test sample 2 (*N* = 204).

	**Decision value**	**Correlation**	**Cronbach’s value after item deletion**	**Factor loading**	**Substandard quantity**
Q4	6.23**	0.55**	0.94	0.47	0
Q5	10.38**	0.65**	0.93	0.61	0
Q6	13.47**	0.67**	0.93	0.61	0
Q7	8.80**	0.57**	0.93	0.52	0
Q8	11.97**	0.71**	0.93	0.66	0
Q9	12.60**	0.69**	0.93	0.65	0
Q10	11.52**	0.71**	0.93	0.70	0
Q11	10.90**	0.70**	0.93	0.66	0
Q12	11.35**	0.63**	0.93	0.52	0
Q13	7.64**	0.58**	0.93	0.52	0
Q14	12.07**	0.67**	0.93	0.64	0
Q15	12.47**	0.71**	0.93	0.68	0
Q16	7.32**	0.56**	0.93	0.60	0
Q17	8.26**	0.60**	0.93	0.66	0
Q18	8.53**	0.63**	0.93	0.62	0
Q19	9.01**	0.63**	0.93	0.58	0
Q20	6.15**	0.50**	0.94	0.49	0
Q21	7.46**	0.52**	0.93	0.60	0
Q22	7.96**	0.54**	0.93	0.62	0
Q23	8.67**	0.64**	0.93	0.67	0
Q24	9.27**	0.62**	0.93	0.68	0
Q25	10.2**	0.66**	0.93	0.69	0
Q26	7.30**	0.53**	0.93	0.50	0
Q27	10.50**	0.65**	0.93	0.65	0
Q28	11.79**	0.67**	0.93	0.62	0
Q29	8.32**	0.54**	0.93	0.49	0
Q30	7.92**	0.53**	0.94	0.47	0
Q31	9.95**	0.65**	0.93	0.59	0
Q32	11.00**	0.64**	0.93	0.61	0
Q33	7.92**	0.53**	0.93	0.61	0
Standard values	≥3.00	≥0.40	≤0.94	≥0.40	

**p < 0.01.

### Exploratory Factor Analysis (EFA) and Retest Reliability

**FIGURE 1:**
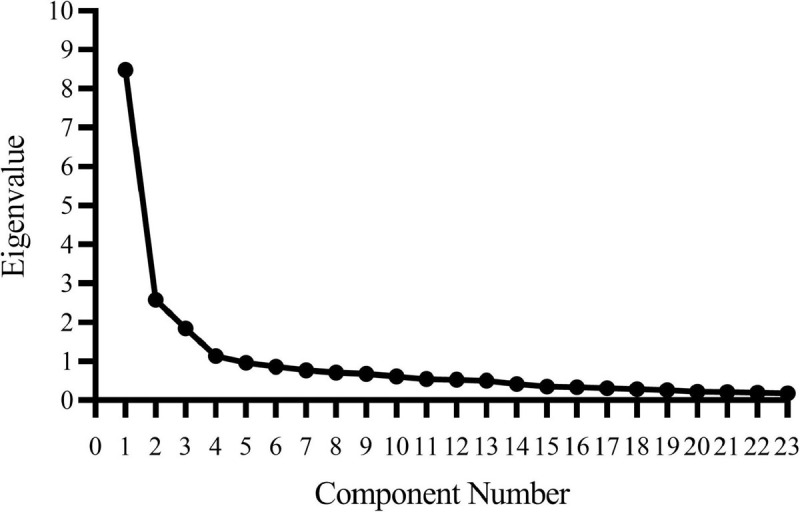
Scree Plot based on EFA of the consumer psychological capital questionnaire.

Bartlett’s test of sphericity and a Kaiser–Meyer–Olkin test applied to the remaining 30 items (χ^2^ = 3606.85, *p* < 0.001, KMO = 0.90) indicated that factor analysis was appropriate (Jang et al., 2009). Because each dimension of psychological capital was interrelated, a principal component analysis with Promax oblique rotation was employed. Based on the theoretical framework, we deleted items with factor loadings less than 0.50 or those that did not meet the requirements of a single dimension to guarantee that the eigenvalues were above 1. We conducted seven EFAs and obtained the four-factor psychological capital scale with 23 questions shown in Supplementary Table S1. The scree plot of the last EFA is provided in Figure 1. The results of the EFA are presented in Table 3. The four factors had a cumulative variance contribution rate of 61.05%. The variance contribution rates of optimism (Factor 1), self-efficacy (Factor 2), hope (Factor 3), and resilience (Factor 4) were 36.87, 11.21, 8.03, and 4.94%, respectively. The factor loadings of all items were greater than 0.60, indicating that the questionnaire had good structural validity. A total of 30 consumers finished the 23 items retest questionnaire (test sample 3); the retest reliability coefficients of optimism, self-efficacy, hope, resilience, and the total scale were 0.82, 0.91, 0.86, 0.88, and 0.92, respectively, suggesting good retest reliability.

**TABLE 3:** Factor loadings of 23 items in test sample 2 (*N* = 204).

	**Optimism (factor 1)**	**Load**	**Self-efficacy (factor 2)**	**Load**	**Hope (factor 3)**	**Load**	**Resilience (factor 4)**	**Load**
	Q30	0.84	Q8	0.84	Q24	0.88	Q16	0.77
	Q32	0.82	Q6	0.82	Q22	0.86	Q19	0.75
	Q29	0.80	Q9	0.78	Q25	0.80	Q13	0.64
	Q28	0.77	Q11	0.70	Q23	0.77	Q12	0.64
	Q31	0.76	Q7	0.68	Q21	0.77		
	Q27	0.73	Q4	0.65				
	Q26	0.60	Q10	0.65				
Eigen values	8.48		2.58		1.85		1.14	
Variance contribution rate (%)	36.87		11.21		8.03		4.94	
Cumulative variance contribution rate (%)	36.87		48.09		56.12		61.05	

### Non-response Bias Analysis and Descriptive Analysis

There is no safe level of response rates below 100%. Particularly for internet surveys, it is difficult to avoid the non-response bias (Alvarez and VanBeselaere, 2005). The study followed up with all 231 consumers who were not included in the final dataset and compared their demographic information with that of 1,076 consumers. The results showed in Table 4 revealed that no significant differences were identified in age, place of residence, education, income, or marital status. In the survey of 1,076 consumers, the mean of their psychological capital scores ranged from 4.23 to 6.33 (standard deviation: 1.01–1.72), and the median was between 4 and 7. In the Spearman’s rank correlation between each item, the largest correlation was found between Q24 and Q25 (correlation = 0.70) while the smallest correlation was between Q29 and Q21 (correlation = 0, see Supplementary Table S2).

**TABLE 4:** Demographic information in invalid respondents (*N* = 231) and respondents (*N* = 1,076).

	**Invalid respondents**	**Respondents**	**Statistics**	***P***
Age [M (P25, P75)]	27(24,30)	27(24,38)	*Z* = −0.88	0.38
Place of resident [N (%)]			χ^2^ = 2.16	0.14
1 = City	141(19.03%)	600(80.97%)		
2 = Rural	90(15.90%)	476(84.10%)		
Education [N (%)]			χ^2^ = 1.12	0.77
1 = Junior high school and below	17(15.89%)	90(84.11%)		
2 = Senior high school or technical secondary school	36(20.00%)	144(8.00%)		
3 = Undergraduate or junior college	127(17.79%)	587(82.21%)		
4 = Postgraduate and above	51(16.67%)	255(83.33%)		
Income [N (%)]			χ^2^ = 2.11	0.72
1 = under 3,000	95(17.50%)	448(82.50%)		
2 = 3,000–5,000	57(16.10%)	297(83.90%)		
3 = 5,000–8,000	45(19.40%)	187(80.60%)		
4 = 8,000–12,000	23(21.10%)	86(78.90%)		
5 = 12,000 and more	11(15.94%)	58(84.06%)		
Marriage status [N (%)]			χ^2^ = 3.35	0.34
1 = Unmarried	138(19.19%)	581(80.81%)		
2 = Married	89(16.01%)	467(83.99%)		
3 = Divorced	2(9.09%)	20(90.91%)		
4 = Widowed	2(20.00%)	8(80.00%)		

*M (P25, P75) indicates the Median and Interquartile Range. N (%) indicates the number and proportion of different respondents’ groups.*

### Confirmatory Factor Analysis (CFA)

Model 1 was a standard four-factor model including self-efficacy, resilience, hope, and optimism. Model 2 was a single-factor model that assumed that all items belonged to one single factor of psychological capital. Model 3 was a three-factor model that combined hope and resilience into one construct, for hope and resilience had a strong positive correlation. The fit indices of models are shown in Table 5. The model fit of the single-factor model (Model 2) was unqualified. The fit indices of Model 1 and Model 3 were not even close to the ideal values. For Model 1, although χ^2^/df, RMSEA, and SRMR obtained values close to excellent, TLI and CFI were 0.87 and 0.85, respectively, which were below the ideal value of 0.90. Low loadings meant that more of the variance in the measure was error variance than an explained variance. Therefore, we deleted items Q12, Q4, Q7, Q21, Q8, Q26, Q27, Q28, and Q22 successively. Meanwhile, any construct of the model should keep at least three items (Hair et al., 2010). The revised model finally included 14 items, which were self-efficacy (Q6, Q9, Q10, Q11), resilience (Q13, Q16, Q19), hope (Q23, Q24, Q25), and optimism (Q29, Q30, Q31, Q32). We freed the residual correlation between Q29 and Q30, and the correlation coefficient was 0.41, which indicated that consumers have a positive attitude toward food operators or manufacturers.

**TABLE 5:** Fit indices of models in the CFA of the consumer psychological capital questionnaire.

	**χ^2^(df.)**	**χ^2^/df**	**RMSEA**	**SRMR**	**CFI**	**TLI**	**Model comparison**	**Δχ^2^**	**Δdf**
Threshold value		≤5.0	≤0.08	≤0.08	≥0.9	≥0.9			
Model 1	1028.5 (224)***	4.59	0.08	0.07	0.87	0.85			
Model 2	2528.9 (230) ***	11.00	0.14	0.11	0.62	0.58	2 vs. 1	1500.40***	6
Model 3	1094.1 (227) ***	4.82	0.08	0.07	0.86	0.84	3 vs. 1	65.60***	3
Model 4′ (final)	234.62 (70) ***	3.35	0.07	0.05	0.95	0.94			
Model 5	265.70 (72) ***	3.69	0.07	0.06	0.94	0.93			
Cross-valid.1 ″	209.94 (70) ***	3.00	0.06	0.05	0.96	0.95			
Cross-valid.2 ″′	248.46 (72) ***	3.45	0.07	0.06	0.95	0.93			

***Indicates significance at the 0.001 level. Model 1: standard four-factor model consisting of self-efficacy, resilience, hope, and optimism;Model 2: single-factor model where 23 items were explained by the psychological capital;Model 3: three-factor model (combined hope and resilience into one factor);Model 4′ (final) indicates the final modified four-factor model with 14 items;Model 5 indicates a second-order model where the psychological capital was set as the second-order factor.Cross-valid1. ″ indicates a cross-validation analysis of Model 4′ (final) using the validation sample (N = 538).Cross-valid2. ″′ indicates a cross-validation analysis of the second-order factor model using the validation sample (N = 538).

**FIGURE 2:**
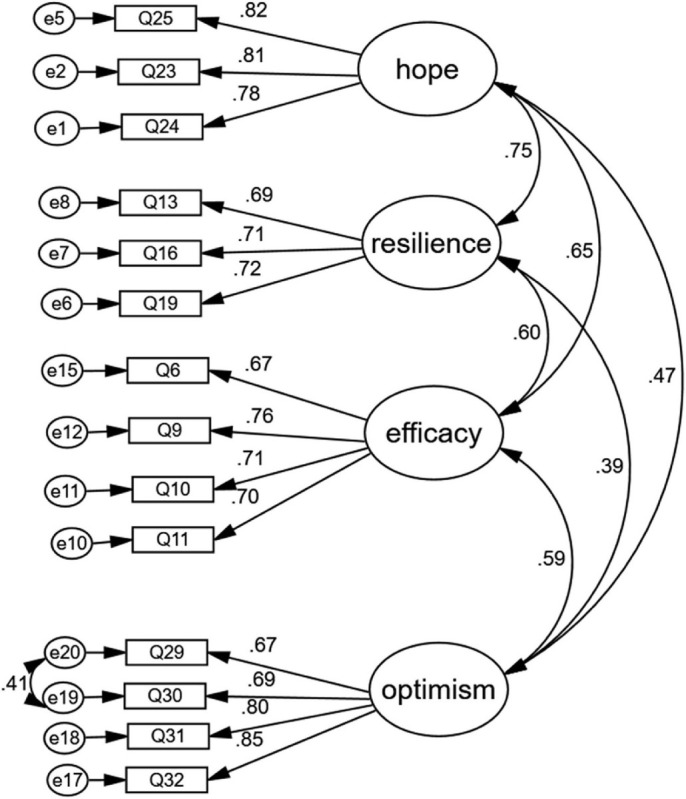
The final 4-factor model of the consumer psychological capital questionnaire in food safety social co-governance.

Model 4 was the final modified four-factor model with 14 items. Figure 2 shows the final 4-factor model of the consumer psychological capital questionnaire in social co-governance of food safety. The model fit of Model 4 was acceptable (see Table 5, χ^2^/df = 3.35, RMSEA = 0.07, SRMR = 0.05, CFI = 0.95, and TLI = 0.94). Generally, χ^2^/df ratios on the order of 3:1 or less are associated with better-fitting models, except in circumstance with larger samples. χ^2^/df smaller than 2.0 is considered very good; between 2.0 and 5.0 is acceptable (Hair et al., 2010). Model 5 was a second-order model based on Model 4; for the correlation coefficients between hope and resilience, hope and self-efficacy were over 0.6. It is necessary to set the psychological capital as a second-order factor to explain efficacy, resilience, hope, and optimism. The model fit of Model 5 was also acceptable (see Table 5, χ^2^/df = 3.69, RMSEA = 0.07, SRMR = 0.06, CFI = 0.94, and TLI = 0.93).

The internal reliability and convergent validity can be found in Table 6. In the modeling sample, the Cronbach’s alpha coefficients of optimism, self-efficacy, hope, resilience, and the total scale were 0.86, 0.80, 0.84, 0.74, and 0.88, respectively. The AVE of hope (0.65), resilience (0.50), self-efficacy (0.51), and optimism (0.57) reached the threshold of 0.5. The CR ranged from 0.75 to 0.85, and the standardized factor loadings ranged from 0.67 and 0.85 with statistical significance. The latent variables in the scale had good convergent validity. The correlations between the latent variables and the square root of AVE of four factors are shown in Table 6. The inter-factor correlation coefficients in the modeling sample were between 0.39 and 0.75. Resilience and hope had a strong correlation, and the remaining dimensions were moderately correlated. Table 7 shows the questionnaire’s discriminant validity. The square root of AVE for optimism, self-efficacy, and hope were 0.76, 0.71, 0.80, respectively, which were greater than the correlation coefficients below the diagonal. The square root of AVE for resilience was 0.71, which was less than the correlation coefficient between resilience and hope of 0.75. A chi-square test was carried out to compare the nested models (see Table 5, Δχ^2^ = 1500.40, *p* < 0.05 between Model 1 and Model 2; Δχ^2^ = 65.60, *p* < 0.05 between Model 1 and Model 3). The overall results showed acceptable discriminant validity for the model, even though the evidence of discriminant validity was not strong.

**TABLE 6:** Internal reliability and convergent validity results of the skew model of model 4 in modeling sample (*N* = 538).

**Factor**	**Item**	**Standardized factor loadings**	**Cronbach’s alpha**	**CR**	**AVE**	**$\sqrt{A\,V\,E}$**
Hope	Q24	0.78***		0.85	0.65	0.80
	Q23	0.81***	0.84			
	Q25	0.82***				
Resilience	Q19	0.72***		0.75	0.50	0.71
	Q16	0.71***	0.74			
	Q13	0.69***				
Self-efficacy	Q11	0.70***		0.80	0.51	0.71
	Q10	0.71***				
	Q9	0.76***	0.80			
	Q6	0.67***				
Optimism	Q32	0.85***		0.84	0.57	0.76
	Q31	0.80***				
	Q30	0.69***	0.86			
	Q29	0.67***				

CR, Construct Reliability; AVE, Average Variance Extracted (AVE); $\sqrt{A\,V\,E}$, the square root of AVE. ***Indicates significance at the 0.001 level.

**TABLE 7:** Descriptive statistics, discriminant validity between latent variables in modeling sample (*N* = 538).

**Factor**	**Mean**	**SD**	**Optimism**	**Self-efficacy**	**Hope**	**Resilience**
Optimism	4.70	1.32	**0.76**			
Self-efficacy	5.07	1.18	0.59	**0.71**		
Hope	6.04	0.95	0.47	0.65	**0.80**	
Resilience	6.16	0.92	0.39	0.60	0.75	**0.71**
Scale	5.41	0.86				

*The values in bold on the diagonal are the square root of AVE for each latent variable.*

The modified four-factor model (Model 4) was validated in the validation sample (cross-validation 1 in Table 5, χ^2^/df = 3.00, RMSEA = 0.06, SRMR = 0.05, CFI = 0.96, TLI = 0.95). The second-order factor model (Model 5) was validated in the validation sample (cross-validation 2 in Table 5, χ^2^/df = 3.45, RMSEA = 0.07, SRMR = 0.06, CFI = 0.95, TLI = 0.93). All the model fit indices reached the acceptable values, indicating that the four-factor model fitted the sample data. Table 7 shows descriptive statistics of four-factor with 14 items. The mean and standard deviation of each dimension varied from 4.70 to 6.16 and from 0.92 to 1.32, respectively. This revealed that consumer psychology was relatively positive regarding the social governance of food safety.

## Discussion

The study aims to develop a tool for measuring consumer psychological capital in food safety social co-governance, with the aim of better understanding and quantifying consumer psychological capital. This is the first consumer-reported questionnaire designed to measure consumer psychological capital in food safety social co-governance. In the development and validation process, support was provided for the factor structure and construct validity of the consumer psychological capital questionnaire. In particular, factor analysis revealed a four-factor structure consisting of self-efficacy, resilience, hope, and optimism. In this study, item screening helped develop the questionnaire with good reliability and validity. The EFA of the consumer psychological capital questionnaire showed that the statistical results were consistent with the theoretical construct and that the four factors contributed 61.05% of the total variance. CFA revealed that the model had adequate convergent validity and acceptable discriminant validity, which was consistent with the construction of consumer psychological capital formed by the EFA. The reliability analysis demonstrated that the dimensions of the questionnaire were highly correlated with items, indicating good reliability of the questionnaire.

### Theoretical Implications

The fit indices of the revised four-factor model (Model 4) and the second-order factor model (Model 5) all reached acceptable values. Therefore, the second-order factor model where the psychological capital was set as a second-order factor was accepted in this study. In other words, it is reasonable to use consumer psychological capital to explain self-efficacy, resilience, hope, and optimism, which provided evidence for hypothesis 1. A previous study also supported the second-order model of psychological capital (Luthans et al., 2007a). According to existing studies, the effect variables of psychological capital mainly include job performance (Avey et al., 2006, 2011), work attitude, and work behavior (Luthans et al., 2005, 2007a), and employee happiness (Avey et al., 2010). Our quantitative study broadened the research directions of psychological capital and focused on a different research field of food safety social co-governance. Whether consumer psychological capital has a positive impact on consumer participation in food safety social governance needs to be examined in future research. In addition, this empirical study of the consumer psychological capital questionnaire supported by reliability and validity test was more convincing to fill in the gaps of conceptual research.

### Managerial Implications

Our quantitative questionnaire focused on the actual problems faced by consumers in food safety social co-governance and developed questionnaire construction from a realistic perspective to analyze the main problems impacting participation in food safety social co-governance. Unsurprisingly, the correlation between all four factors was positive, which provided evidence for hypothesis 2. This is consistent with the conclusions of previous studies (Luthans et al., 2005; Luthans and Youssef-Morgan, 2017). The self-efficacy dimension captures consumers who have a strong sense of responsibility for food safety and knowledge of how to report food safety issues in a variety of ways. Consumers with high self-efficacy had higher resilience, hope, and optimism in the process of food safety social co-governance. The optimism dimension involves consumers’ positive attitudes toward other consumers, food business operators, media, and food regulators. Optimistic individuals always perceive a greater chance for success; most of them trust the food safety department, other consumers, the media, and food enterprises to perform well. Confident consumers have high self-efficacy; it seems that they can deal with challenging food safety incidents. The hope dimension involves consumers knowing the importance of participating in food safety and striving to fully participate in food safety supervision. Having hope helps consumers pursue multiple pathways to achieve their goals. Resilience offers a helping hand to allow for recovery from setbacks when people face food safety problems. Consumers can report food safety issues bravely and learn a lesson from it.

From our questionnaire survey results, the following main recommendations can be made regarding how to strengthen consumer psychological capital. First, food safety departments should publicize basic information about food safety and rights protections through online or offline channels to make consumers more familiar with food safety knowledge and enhance their self-efficacy. Second, according to the severity of the problems reported by consumers, a corresponding incentive mechanism should be established to mitigate the opportunity costs, such as time and money, encountered by consumers in the process of safeguarding their rights. This would have a positive effect on fostering consumer resilience. Third, the government should enhance consumer praise and publicize consumers’ reports of food safety issues, encourage consumers to establish a sense of responsibility for food safety, and make consumers understand that actively participating in food safety social co-governance protects not only their rights but also those of other consumers. Thus, consumers will have a higher level of hope for food safety social co-governance. Fourth, severely punishing food operators who are aware of and violate the law would not only improve the government’s credibility but also increase consumer trust in the government and enterprises. Consumers’ optimistic attitudes are more conducive to promoting social harmony.

### Research Limitations

This study has several limitations that should be noted. Although the online survey was convenient, simple, cost-effective, and wide in coverage, the representativeness of the sample could be improved. Further studies are recommended to evaluate the scale’s factor structure across varied respondents. In addition, the selection bias and non-response bias are inevitable in an internet survey. We examined whether demographic information was statistically significant between 231 and 1,076 consumers and randomly allocated the 1,076 consumers into the modeling sample and validation sample to validate the questionnaire’s validity, but we still need to collect random investigated data to minimize the selection bias and non-response bias. Due to the lack of domestic and foreign research studies on psychological capital enabling consumers to participate in food safety social co-governance, no mature scale is available for reference. Therefore, the criterion calibration validity of the self-compiled scale in this study has yet to be verified.

### Suggestions for Further Studies

This research simply provided a reliable tool to measure consumer psychological capital in food safety social co-governance. More quantitative statistical analysis verifying whether psychological capital is positively correlated with consumer participation is still needed in future studies. The questionnaire also motivates us to explore and measure the role of psychological capital in the relationship with government or food enterprises. In other words, cultivating the psychological capital of government or food enterprises is a flexible approach to food safety management. We contend that psychological capital has a positive effect on solving food safety problems for government or enterprises, and will collect more data to confirm this view.

## Conclusion

In conclusion, the consumer psychological capital questionnaire developed in this exploratory study has good reliability and convergent validity, and acceptable discriminant validity. There were 14 items in the questionnaire, including four factors: optimism, self-efficacy, hope, and resilience. The rationality of this consumer psychological capital questionnaire was supported by the EFA and CFA. This questionnaire can be used as an effective tool to measure consumer participation in food safety social co-governance in further research.

## Data Availability Statement

The raw data supporting the conclusions of this article will be made available by the authors, without undue reservation.

## Ethics Statement

The studies involving human participants were reviewed and approved by the Ethics Committee of Chongqing Medical University. The patients/participants provided their written informed consent to participate in this study.

## Author Contributions

BP and CM designed this study. LS, XG, MW, LY, and YW constructed the original concepts and assisted in data collection. CM performed the study, analyzed the data, and drafted the manuscript. BP reviewed and revised the manuscript. All authors contributed to the article and approved the submitted version.

## Conflict of Interest

The authors declare that the research was conducted in the absence of any commercial or financial relationships that could be construed as a potential conflict of interest.
