# Supplementary material for: Development and Validation of a Questionnaire on Consumer Psychological Capital in Food Safety Social Co-governance
Source: Front Psychol. 2021 Jan 7;11:584810. doi: 10.3389/fpsyg.2020.584810 (PMC7817848; doi:10.3389/fpsyg.2020.584810)
Supplement: Supplementary file 1 [file Table_1.DOCX]

Table S1 23 psychological capital questions in the formal questionnaire

|  |  | strongly disagree | relatively  disagree | somewhat  disagree | unclear | somewhat  agree | relatively  agree | strongly agree |
| --- | --- | --- | --- | --- | --- | --- | --- | --- |
| Self-efficacy | Q4. I can positively learn about ways to protect food safety through mobile phones, TV, or offline publicity. |  |  |  |  |  |  |  |
|  | Q6. If I buy expired, spoiled, mildewed, or toxic food, I can quickly complain and report to the relevant department by telephone, letter, network, etc. |  |  |  |  |  |  |  |
|  | Q7. I know how to find online channels and methods for safeguarding consumer rights. |  |  |  |  |  |  |  |
|  | Q8. I can use QQ, WeChat, Weibo, and other network platforms to play the role of a food safety supervisor better. |  |  |  |  |  |  |  |
|  | Q9. If I find that illegal businesses have illegal operations, I dare to report it to the relevant departments. |  |  |  |  |  |  |  |
|  | Q10. If I encounter a serious food safety issue such as food poisoning, I can report it to the relevant department in time. |  |  |  |  |  |  |  |
|  | Q11. I think I have a strong sense of responsibility for food safety. |  |  |  |  |  |  |  |
| Resilience | Q12. I have zero tolerance for unsafe and unqualified food. |  |  |  |  |  |  |  |
|  | Q13. If I find out that the food has expired or deteriorated before buying it, I will take the initiative to submit it to the merchant. |  |  |  |  |  |  |  |
|  | Q16. If I buy expired, spoiled, moldy, or poisonous food, I will inform the people around me in time. |  |  |  |  |  |  |  |
|  | Q19. When buying food, I will try to understand its safety as much as possible by checking the shelf life, color, and taste. |  |  |  |  |  |  |  |
| Hope | Q21. If I report a problem of food, I hope to receive a timely and fair response from the relevant department. |  |  |  |  |  |  |  |
|  | Q22. I think participating in food safety governance can not only protect your legal rights and health but also protect the rights and health of other consumers. |  |  |  |  |  |  |  |
|  | Q23. I think that I have a sense of social responsibility, prompting me to participate in food safety governance and give full play to food safety supervision. |  |  |  |  |  |  |  |
|  | Q24. I think that active participation in food safety governance and being a good food safety supervisor is good for the food safety social co-governance. |  |  |  |  |  |  |  |
|  | Q25. I understand that I am the most personal experience of safe food and should actively participate in food safety governance. |  |  |  |  |  |  |  |
| Optimism | Q26. I believe that most of the foods circulating in the market are safe. |  |  |  |  |  |  |  |
|  | Q27. I believe that most consumers can play a role in food supervision. |  |  |  |  |  |  |  |
|  | Q28. I believe that most consumers can positively report, complain about unsafe food that they encounter. |  |  |  |  |  |  |  |
|  | Q29. For manufacturers or brands that have exposed food safety incidents, I believe they can take corresponding responsibilities and correct them. |  |  |  |  |  |  |  |
|  | Q30. I believe that most food business operators have an attitude of safety-first and benefit second. |  |  |  |  |  |  |  |
|  | Q31. I believe that the media can accurately and timely disseminate food safety incidents. |  |  |  |  |  |  |  |
|  | Q32. I think food regulators will promptly deal with food safety issues reported by consumers |  |  |  |  |  |  |  |

Table S2 Descriptive analysis and Spearman correlation coefficients of 23 items in sample test 4 (N=1076)

| Item | Mean | SD | M | 1 | 2 | 3 | 4 | 5 | 6 | 7 | 8 | 9 | 10 | 11 | 12 | 13 | 14 | 15 | 16 | 17 | 18 | 19 | 20 | 21 | 22 | 23 |
| --- | --- | --- | --- | --- | --- | --- | --- | --- | --- | --- | --- | --- | --- | --- | --- | --- | --- | --- | --- | --- | --- | --- | --- | --- | --- | --- |
| Q4 | 4.93 | 1.36 | 5 | 1.00 |  |  |  |  |  |  |  |  |  |  |  |  |  |  |  |  |  |  |  |  |  |  |
| Q6 | 4.94 | 1.60 | 5 | 0.51 | 1.00 |  |  |  |  |  |  |  |  |  |  |  |  |  |  |  |  |  |  |  |  |  |
| Q7 | 4.67 | 1.59 | 5 | 0.48 | 0.48 | 1.00 |  |  |  |  |  |  |  |  |  |  |  |  |  |  |  |  |  |  |  |  |
| Q8 | 4.90 | 1.52 | 5 | 0.51 | 0.49 | 0.56 | 1.00 |  |  |  |  |  |  |  |  |  |  |  |  |  |  |  |  |  |  |  |
| Q9 | 4.73 | 1.54 | 5 | 0.37 | 0.56 | 0.44 | 0.50 | 1.00 |  |  |  |  |  |  |  |  |  |  |  |  |  |  |  |  |  |  |
| Q10 | 5.50 | 1.39 | 6 | 0.36 | 0.45 | 0.38 | 0.40 | 0.55 | 1.00 |  |  |  |  |  |  |  |  |  |  |  |  |  |  |  |  |  |
| Q11 | 4.81 | 1.45 | 5 | 0.34 | 0.42 | 0.41 | 0.45 | 0.55 | 0.52 | 1.00 |  |  |  |  |  |  |  |  |  |  |  |  |  |  |  |  |
| Q12 | 4.93 | 1.67 | 5 | 0.27 | 0.35 | 0.30 | 0.32 | 0.44 | 0.39 | 0.48 | 1.00 |  |  |  |  |  |  |  |  |  |  |  |  |  |  |  |
| Q13 | 5.84 | 1.37 | 6 | 0.27 | 0.35 | 0.24 | 0.25 | 0.37 | 0.43 | 0.36 | 0.37 | 1.00 |  |  |  |  |  |  |  |  |  |  |  |  |  |  |
| Q16 | 6.19 | 1.07 | 6 | 0.25 | 0.31 | 0.27 | 0.24 | 0.30 | 0.40 | 0.32 | 0.34 | 0.55 | 1.00 |  |  |  |  |  |  |  |  |  |  |  |  |  |
| Q19 | 6.14 | 1.10 | 6 | 0.24 | 0.25 | 0.25 | 0.25 | 0.25 | 0.36 | 0.33 | 0.32 | 0.47 | 0.56 | 1.00 |  |  |  |  |  |  |  |  |  |  |  |  |
| Q21 | 6.33 | 1.07 | 7 | 0.23 | 0.20 | 0.22 | 0.21 | 0.20 | 0.34 | 0.22 | 0.24 | 0.41 | 0.48 | 0.51 | 1.00 |  |  |  |  |  |  |  |  |  |  |  |
| Q22 | 6.29 | 1.06 | 7 | 0.28 | 0.26 | 0.23 | 0.27 | 0.26 | 0.36 | 0.32 | 0.30 | 0.45 | 0.51 | 0.54 | 0.62 | 1.00 |  |  |  |  |  |  |  |  |  |  |
| Q23 | 5.82 | 1.16 | 6 | 0.27 | 0.33 | 0.26 | 0.33 | 0.41 | 0.39 | 0.46 | 0.40 | 0.44 | 0.41 | 0.44 | 0.40 | 0.58 | 1.00 |  |  |  |  |  |  |  |  |  |
| Q24 | 6.18 | 1.01 | 6 | 0.28 | 0.29 | 0.23 | 0.27 | 0.27 | 0.34 | 0.32 | 0.32 | 0.41 | 0.47 | 0.48 | 0.48 | 0.67 | 0.66 | 1.00 |  |  |  |  |  |  |  |  |
| Q25 | 5.92 | 1.15 | 6 | 0.26 | 0.31 | 0.22 | 0.30 | 0.37 | 0.37 | 0.42 | 0.39 | 0.41 | 0.42 | 0.44 | 0.41 | 0.59 | 0.67 | 0.70 | 1.00 |  |  |  |  |  |  |  |
| Q26 | 5.21 | 1.34 | 6 | 0.23 | 0.25 | 0.22 | 0.28 | 0.24 | 0.21 | 0.23 | 0.20 | 0.22 | 0.18 | 0.17 | 0.16 | 0.23 | 0.26 | 0.24 | 0.30 | 1.00 |  |  |  |  |  |  |
| Q27 | 5.08 | 1.41 | 5 | 0.26 | 0.35 | 0.26 | 0.33 | 0.36 | 0.31 | 0.37 | 0.30 | 0.26 | 0.26 | 0.24 | 0.17 | 0.29 | 0.39 | 0.29 | 0.41 | 0.55 | 1.00 |  |  |  |  |  |
| Q28 | 4.97 | 1.48 | 5 | 0.28 | 0.40 | 0.27 | 0.36 | 0.44 | 0.38 | 0.39 | 0.33 | 0.34 | 0.30 | 0.26 | 0.19 | 0.28 | 0.38 | 0.30 | 0.39 | 0.46 | 0.69 | 1.00 |  |  |  |  |
| Q29 | 4.57 | 1.57 | 4 | 0.18 | 0.25 | 0.19 | 0.26 | 0.29 | 0.20 | 0.24 | 0.19 | 0.17 | 0.09 | 0.12 | 0.00 | 0.07 | 0.26 | 0.12 | 0.20 | 0.39 | 0.47 | 0.52 | 1.00 |  |  |  |
| Q30 | 4.23 | 1.72 | 4 | 0.20 | 0.24 | 0.23 | 0.29 | 0.30 | 0.19 | 0.27 | 0.20 | 0.13 | 0.13 | 0.12 | 0.00 | 0.05 | 0.20 | 0.09 | 0.20 | 0.45 | 0.50 | 0.51 | 0.67 | 1.00 |  |  |
| Q31 | 4.88 | 1.54 | 5 | 0.31 | 0.34 | 0.31 | 0.35 | 0.38 | 0.31 | 0.34 | 0.25 | 0.24 | 0.24 | 0.20 | 0.12 | 0.18 | 0.28 | 0.22 | 0.27 | 0.38 | 0.48 | 0.54 | 0.52 | 0.58 | 1.00 |  |
| Q32 | 4.97 | 1.42 | 5 | 0.33 | 0.41 | 0.35 | 0.38 | 0.42 | 0.35 | 0.37 | 0.29 | 0.29 | 0.25 | 0.25 | 0.15 | 0.23 | 0.35 | 0.28 | 0.32 | 0.45 | 0.52 | 0.54 | 0.55 | 0.58 | 0.68 | 1.00 |
